# Age-differential sexual dimorphisms in CHD8-S62X-mutant mouse synapses and transcriptomes

**DOI:** 10.3389/fnmol.2023.1111388

**Published:** 2023-02-16

**Authors:** Soo Yeon Lee, Hanseul Kweon, Hyojin Kang, Eunjoon Kim

**Affiliations:** ^1^Department of Biological Sciences, Korea Advanced Institute for Science and Technology (KAIST), Daejeon, Republic of Korea; ^2^Center for Synaptic Brain Dysfunctions, Institute for Basic Science (IBS), Daejeon, Republic of Korea; ^3^Division of National Supercomputing, Korea Institute of Science and Technology Information, Daejeon, Republic of Korea

**Keywords:** autism spectrum disorder, chromatin remodeling, sexual dimorphism, age dependence, synapse, transcriptome

## Abstract

*Chd8^+/N2373K^* mice with a human C-terminal-truncating mutation (N2373K) display autistic-like behaviors in juvenile and adult males but not in females. In contrast, *Chd8^+/S62X^* mice with a human N-terminal-truncating mutation (S62X) display behavioral deficits in juvenile males (not females) and adult males and females, indicative of age-differential sexually dimorphic behaviors. Excitatory synaptic transmission is suppressed and enhanced in male and female *Chd8^+/S62X^* juveniles, respectively, but similarly enhanced in adult male and female mutants. ASD-like transcriptomic changes are stronger in newborn and juvenile (but not adult) *Chd8^+/S62X^* males but in newborn and adult (not juvenile) *Chd8^+/S62X^* females. These results point to age-differential sexual dimorphisms in *Chd8^+/S62X^* mice at synaptic and transcriptomic levels, in addition to the behavioral level.

## Introduction

Mutations of *CHD8*, encoding a chromatin remodeler, have been extensively associated with autism spectrum disorders (ASD; Bernier et al., 2014; Barnard et al., 2015; Dingemans et al., 2022; Zhou et al., 2022). Human individuals carrying *CHD8* mutations show a strong male–female ratio (~85:15; Stessman et al., 2017), suggesting that CHD8 could be one of the ideal targets for studying the mechanisms underlying male–female differences in ASD.

Previous studies on *Chd8*-mutant mice and human neurons suggested multiple mechanisms that may underlie CHD8-related brain deficits (Sugathan et al., 2014; Cotney et al., 2015; Wang et al., 2015; Breuss and Gleeson, 2016; Durak et al., 2016; Katayama et al., 2016; Gompers et al., 2017; Platt et al., 2017; Wang et al., 2017; Andreae and Basson, 2018; Jung et al., 2018; Suetterlin et al., 2018; Wade et al., 2018; Xu et al., 2018; Zhao et al., 2018; Hulbert et al., 2020; Jimenez et al., 2020; Sood et al., 2020; Cherepanov et al., 2021; Ding et al., 2021; Ellingford et al., 2021; Hurley et al., 2021; Kawamura et al., 2021; Kweon et al., 2021; Weissberg and Elliott, 2021; Chen et al., 2022; Coakley-Youngs et al., 2022; Dong et al., 2022; Haddad Derafshi et al., 2022; Jimenez et al., 2022; Kerschbamer et al., 2022; Lee et al., 2022; Paulsen et al., 2022; Thudium et al., 2022; Tu et al., 2022; Villa et al., 2022; Yu et al., 2022; Hayot et al., 2023). However, mechanisms underlying the male–female differences in CHD8-related ASD are poorly understood (Jung et al., 2018; Cherepanov et al., 2021; Yu et al., 2022).

*Chd8^+/N2373K^* mice that carry a human mutation that leads to a C-terminal protein truncation (N2373K; O’Roak et al., 2012; Merner et al., 2016) display male-preponderant behavioral deficits as juveniles and adults. In contrast, *Chd8^+/S62X^* mice expressing CHD8-S62X proteins with N-terminal truncation (O’Roak et al., 2012) display behavioral deficits in juvenile males (not females) and adult males and females (Lee et al., 2022), indicative of age-differential sexually dimorphic behaviors. Here, we characterized and compared synaptic and transcriptomic phenotypes in male and female *Chd8^+/S62X^* mice at juvenile and adult stages, which led us to find age-differential sexual dimorphisms at synaptic and transcriptomic levels additional to the behavioral level.

## Materials and methods

### Animals

*Chd8^+/S62X^* mice have been recently reported (Lee et al., 2022). Mice were maintained at the Korea Advanced Institute of Science and Technology (KAIST) mouse facility (12-h light–dark cycle).

### Electrophysiology

Electrophysiology was performed using mouse brain slices containing the dorsal hippocampus, a brain region associated with ASD (Schumann et al., 2004), from juvenile mice at P21–28 and adult mice at 9–18 weeks. Isoflurane-anesthetized mice were used to dissect brains, which were placed in a chamber containing dissection buffer (In mM: 212 sucrose, 25 NaHCO_3_, 1.25 NaH_2_PO_4_, 5 KCl, 3.5 MgCl_2_, 0.5 CaCl_2_, 10 D-glucose, 2 sodium pyruvate, 1.2 sodium ascorbate, 95% O_2_, and 5% CO_2_) and used to obtain sagittal slices (300 μm; ~1–2 slices/mice) using a vibratome; exact numbers of mice and neurons used for each experiment are described in the figure legends. Slices containing the dorsal hippocampus were recovered in the artificial cerebrospinal fluid (aCSF; in mM: 125 NaCl, 25 NaHCO_3_, 1.25 NaH_2_PO_4_, 2.5 KCl, 1.3 MgCl_2_, 2.5 CaCl_2_, 10 D-glucose) at 32°C for 30 and 60 min (juvenile and adult, respectively) and at 20°C–25°C for 30 min, followed by a transfer to a recording chamber at 28°C with circulating aCSF. sEPSCs were measured in the presence of picrotoxin (60 μM; Sigma), and mEPSCs were measured in the presence of picrotoxin (60 μM) and tetrodotoxin (0.5 μM; Tocris). sIPSCs were measured in the presence of NBQX (10 μM; Tocris) and D-AP5 (50 μM; Tocris). mIPSCs were measured in the presence of NBQX (10 μM), D-AP5 (50 μM), and tetrodotoxin (0.5 μM). Recording pipettes (2.0~3.5 MΩ resistance) were pulled from borosilicate capillaries (thin-walled, 30-0065, Harvard Apparatus) using a two-step vertical micropipette puller (PC-10, Narishige). The pipette-filling solution for sEPSC and mEPSC recordings contained (in mM) 117 CsMeSO_4_, 8 NaCl, 10 TEACl, 10 EGTA, 10 HEPES, 4 Mg-ATP, 0.3 Na-GTP, and 5 QX-314, and the solution for sIPSC and mIPSC recordings contained (in mM) 115 CsCl, 8 NaCl, 10 TEACl, 10 EGTA, 10 HEPES, 4 Mg-ATP, 0.3 Na-GTP, and 5 QX-314. For giga sealing, neurons were approached with the internal solution-filled pipette and gently ruptured through suction. Membrane voltages were held at −70 mV, and recordings were obtained after cells were perfused with aCSF and stabilized (2 min). Signals were filtered (2 kHz) and digitized (10 kHz) using Multiclamp 700B amplifier and Digidata 1,550 digitizer (Molecular Devices). The access resistance, checked before and after recordings, was used to exclude data when it is >20 MΩ. Acquired data were analyzed using Clampfit 10 (Molecular Devices).

### RNA-Seq analysis

Three mice aged P0, P25, and P80 were used for each group (heterozygous, wildtype, male, female). Brains were quickly dissected and deep-freezed in RNAlater solution (Ambion) to stabilize RNAs. RNA extraction, library preparation, cluster generation, and sequencing were conducted by Macrogen. Sequencing was performed with an average read depth of 70 to 90 million reads at paired-ends (2 × 101 bp) using an Illumina HiSeq 4000 (Illumina) *via* Macrogen Inc. Transcript abundance was estimated in pseudo-mapping-based mode for the Mus musculus genome (GRCm38) using Salmon (v1.1.0; Patro et al., 2017). Differential gene expression analysis was performed using R/Bioconductor DEseq2 (v1.26.0; Love et al., 2014) by importing the estimated abundance data into R (v.3.5.3) using the tximport (Soneson et al., 2015) package. The *p*-values were adjusted for multiple testing with the Benjamini–Hochberg correction. A threshold for differentially expressed genes was used as a gene with an adjusted *p*-value of less than 0.05. We did not attempt RT-qPCR validation of RNA-Seq results considering that RNA-Seq results are usually well correlated with RT-qPCR results and that our study extracts most of the biological functions through GSEA (not DEG analysis), which uses a large number of genes with small changes (Subramanian et al., 2005) that are difficult to validate by RT-qPCR.

### Gene set enrichment analysis

Fold changes and adjusted *p*-values from differential gene expression (DEG) analysis were used to perform Gene Set Enrichment Analysis (GSEA; Subramanian et al., 2005). GSEA allows us to capture if the expressions of genes in a specific gene set are changed in a consistent direction, although each might not be significant enough to be counted as differentially expressed genes. The GSEA software (gsea2-2.2.4.jar; http://software.broadinstitute.org/gsea) was used to obtain enrichment scores. All expressed genes were ranked by the sign of fold change multiplied by –log10 (*p*-value). 1,000 permutations and a classic scoring scheme were used, as recommended by the program. Gene sets used in this study included those from the Molecular Signature Database (MSigDB v7.0; http://software.broadinstitute.org/gsea/msigdb) and ASD-related gene sets from previous studies (gene sets that are either upregulated or downregulated in ASD and ASD-risk gene sets; see the Results section below for details; Voineagu et al., 2011; Iossifov et al., 2014; Werling et al., 2016; Yang et al., 2018). GSEA results were visualized using EnrichmentMap App in Cytoscape 3.8.0. The RNA-Seq results are deposited in the NCBI GEO (National Center for Biotechnology Information, Gene Expression Omnibus) repository as GSE167053.

## Statistics

Outlying data were excluded using ROUT test (Q = 1%). For male–female comparisons, two-way ANOVA (sex and genotype factors) with assumed normality was used. Graphpad Prism 9 and SigmaPlot 12.0 programs were used for statistical analyses. Statistical details are shown in Supplementary Table S1.

## Results

### Increased excitatory synaptic transmission in adult *Chd8^+/S62X^* males and females

To determine whether the similarly altered behaviors in adult *Chd8^+/S62X^* males and females (Lee et al., 2022) accompany synaptic dysfunctions, we measured synaptic transmissions in the adult hippocampus, a brain region associated with ASD (Schumann et al., 2004).

**Figure 1:**
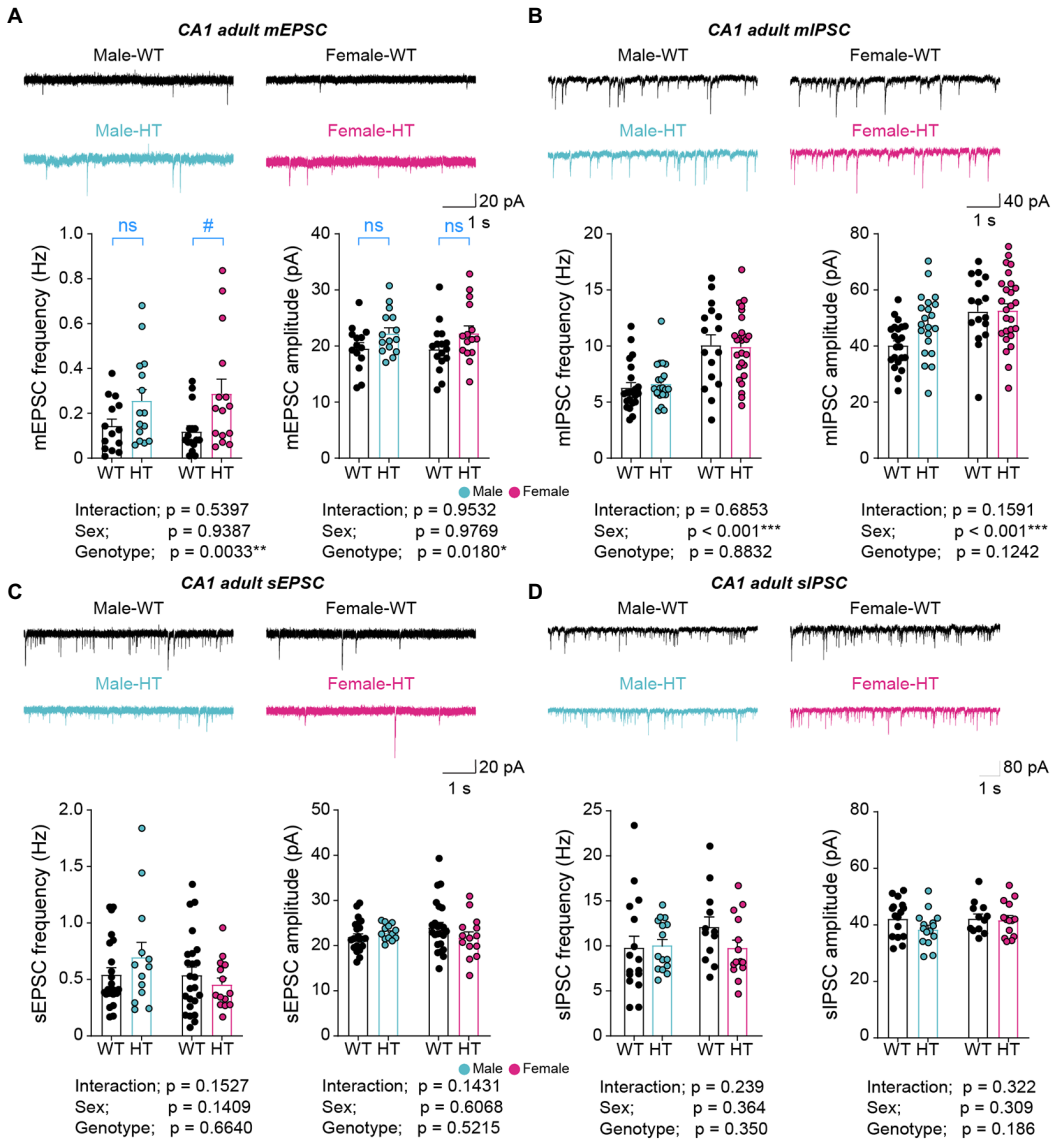
Increased excitatory synaptic transmission in adult Chd8+/S62X males and females. **(A)** mEPSCs in CA1 pyramidal neurons in adult *Chd8^+/S62X^* males and females (15–18 weeks). (*n* = 14 neurons from 3 mice [male-WT], 15/4 [male-HT], 16/4 [female-WT], and 15/3 [female-HT], *p < 0.05, **p < 0.01, two-way ANOVA, ^#^*p* < 0.05, ns, not significant, Mann–Whitney *U-*test). **(B)** mIPSCs in CA1 pyramidal neurons in adult *Chd8^+/S62X^* males and females (13–16 weeks). (*n* = 22/4 [male-WT], 21/5 [male-HT], 16/3 [female-WT], and 26/4 [female-HT], ***p < 0.001, two-way ANOVA). **(C)** sEPSCs in CA1 pyramidal neurons in adult *Chd8^+/S62X^* males and females (13–16 weeks). (*n* = 14/3 [male-WT], 15/4 [male-HT], 16/4 [female-WT], and 15/3 [female-HT], two-way ANOVA). **(D)** sIPSCs in CA1 pyramidal neurons in adult *Chd8^+/S62X^* males and females (13–16 weeks). (*n* = 16/3 [male-WT], 15/3 [male-HT], 12/3 [female-WT], and 14/3 [female-HT], two-way ANOVA).

Adult (13–18 weeks) male and female *Chd8^+/S62X^* mice showed significant increases in miniature excitatory postsynaptic currents (mEPSCs) in CA1 hippocampal neurons compared with WT mice, as supported by genotype differences in mEPSC frequency and amplitude in two-way ANOVA analysis (Figure 1A; Supplementary Table S1). However, an additional Mann–Whitney *U-*test performed, based on the insignificant sex x genotype interaction in the two-way ANOVA, indicated a genotype difference in females but not in males in the frequency and no genotype difference in males or females in the amplitude. In contrast to these excitatory synaptic changes, there were no alterations in miniature inhibitory postsynaptic currents (mIPSCs) in *Chd8^+/S62X^* males or females compared with WT mice, although females showed greater mIPSC frequency and amplitude compared with males (Figure 1B).

The excitatory synaptic differences, however, became insignificant when spontaneous excitatory postsynaptic currents (sEPSCs) were recorded by omitting the action-potential blocker tetrodotoxin in recording solutions to allow network activity (Figure 1C). Spontaneous inhibitory postsynaptic currents (sIPSCs) were also not different between genotypes (Figure 1D).

These results collectively suggest that the CHD8-S62X mutation enhances excitatory, but not inhibitory, synaptic transmission in adult male and female mutant mice, and that network activity compensates for the mutation-induced excitatory synaptic changes.

### Opposite changes in excitatory synaptic transmission in juvenile *Chd8^+/S62X^* males and females

Given the stronger mother-seeking/attachment behavior in juvenile male *Chd8^+/S62X^* mice (Lee et al., 2022), we next tested whether juvenile (postnatal day [P] 22–28) *Chd8^+/S62X^* males and females show any differential changes in hippocampal mEPSCs or mIPSCs.

**Figure 2:**
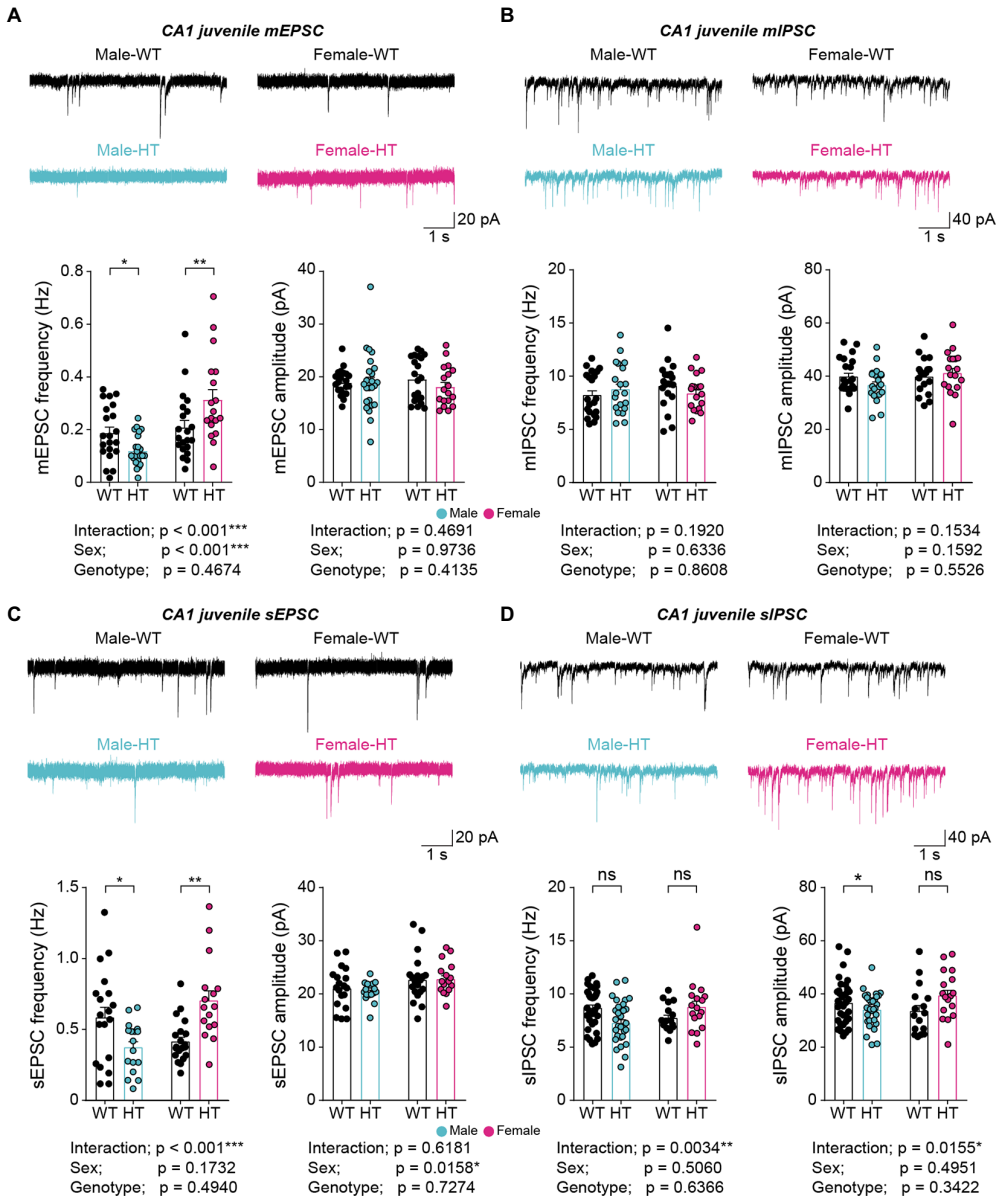
Opposite changes in excitatory synaptic transmission in juvenile Chd8+/S62X males and females. **(A)** mEPSCs in CA1 pyramidal neurons in juvenile *Chd8^+/S62X^* males and females (P25–28). (*n* = 21, 6 [male-WT], 24, 6 [male-HT], 21, 4 [female-WT], and 18, 4 [female-HT], *p < 0.05, **p < 0.01, ***p < 0.001, two-way ANOVA with Holm-Sidak test). **(B)** mIPSCs in CA1 pyramidal neurons in juvenile *Chd8^+/S62X^* males and females (P22–28). (*n* = 21, 4 [male-WT], 19, 4 [male-HT], 20, 4 [female-WT], and 18, 4 [female-HT], two-way ANOVA). **(C)** sEPSCs in CA1 pyramidal neurons in juvenile *Chd8^+/S62X^* males and females (P22–28). (*n* = 19, 4 [male-WT], 17, 4 [male-HT], 20, 4 [female-WT], and 16, 3 [female-HT], *p < 0.05, **p < 0.01, ***p < 0.001, two-way ANOVA with Holm-Sidak test). **(D)** sIPSCs in CA1 pyramidal neurons in juvenile *Chd8^+/S62X^* males and females (P22–28). (*n* = 35, 5 [male-WT], 31, 5 [male-HT], 16, 3 [female-WT], and 17, 3 [female-HT], *p < 0.05, **p < 0.01, ns, not significant, two-way ANOVA with Holm-Sidak test).

Intriguingly, juvenile male and female *Chd8^+/S62X^* mice showed opposite changes in mEPSCs, but not mIPSCs, compared with WT mice, with the frequency of mEPSCs decreasing in males but increasing in females; the amplitudes of mEPSCs were unaffected in either sex (Figures 2A,B). In addition, allowing network activity during recordings by omitting tetrodotoxin did not affect the oppositely changed mEPSC frequencies observed in juvenile male and female *Chd8^+/S62X^* mice (Figures 2C,D), in contrast to the abovementioned compensatory effects of network activity on mEPSCs in adult *Chd8^+/S62X^* mice. However, a moderate decrease in sIPSC amplitude (not frequency) was observed in male but not female *Chd8^+/S62X^* mice in the presence of network activity (Figure 2D).

Therefore, the CHD8-S62X mutation results in the opposite, or sexually dimorphic, changes in excitatory, but not inhibitory, synaptic transmissions that are resistant to network correction in the juvenile mice, which contrasts with the sensitivity of the excitatory synaptic change to network correction in adult mutant mice.

### Age-differential ASD-like transcriptomic changes in male and female *Chd8^+/S62X^* mice

The results recently reported (Lee et al., 2022) and mentioned above indicate largely similar synaptic and behavioral phenotypes in adult male and female mutant mice but sexually dimorphic synaptic changes and male-preponderant behavioral deficits in juvenile mutant males and females. To better understand the mechanisms underlying these results, we performed a longitudinal analysis of transcriptomic changes in the whole brain of P0 (newborn), P25 (juvenile), and P80 (adult) *Chd8^+/S62X^* males and females (see Supplementary Table S2 for raw RNA-Seq data).

**Figure 3:**
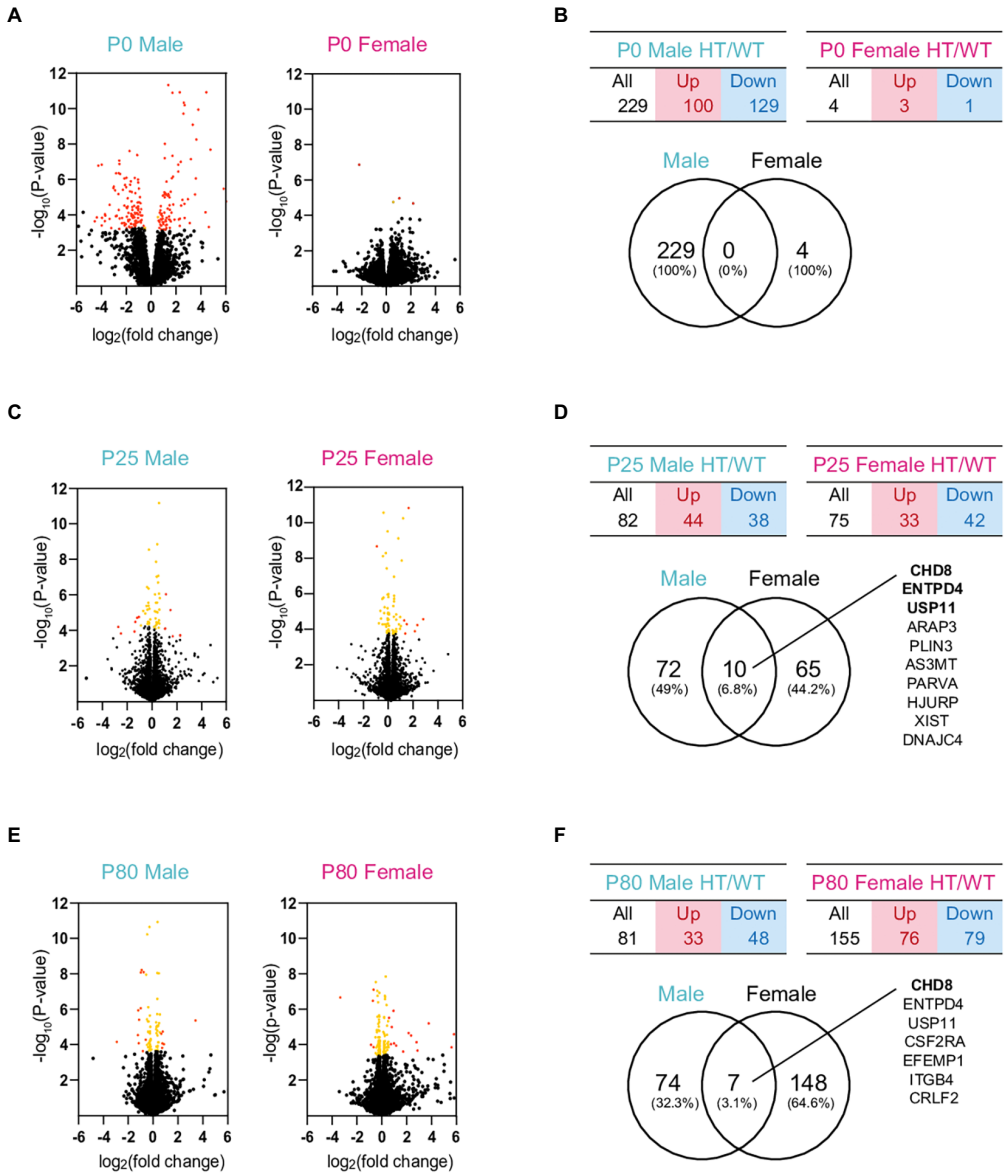
DEG analyses of RNA-Seq results from P0, P25, and P80 male and female HT/WT transcripts. **(A–F)** Volcano plots, tables, and Venn diagrams summarizing the DEGs from P0, P25, and P80 male and female HT/WT transcripts. Orange, genes with adjusted *p*-value < 0.05; red, genes with adjusted *p*-value < 0.05 + |FC| > 1.5 (see Supplementary Table S3 for further details on DEGs). Note that the number of overlapping DEGs between males and females is small.

Analyses of the RNA-Seq results with a focus on differentially expressed genes (DEGs) did not reveal detectable changes in biological functions, likely because of the small numbers of DEGs in P0, P25, and P80 *Chd8^+/S62X^* males and females (Figures 3A–F; see Supplementary Table S3 for the complete list of DEGs). We thus performed a gene set enrichment analysis (GSEA), a method optimized to identify biological functions that depend on a large number of genes with small, but coordinated, changes rather than by a small number of genes with large changes above artificial cutoffs.^1^

**Figure 4:**
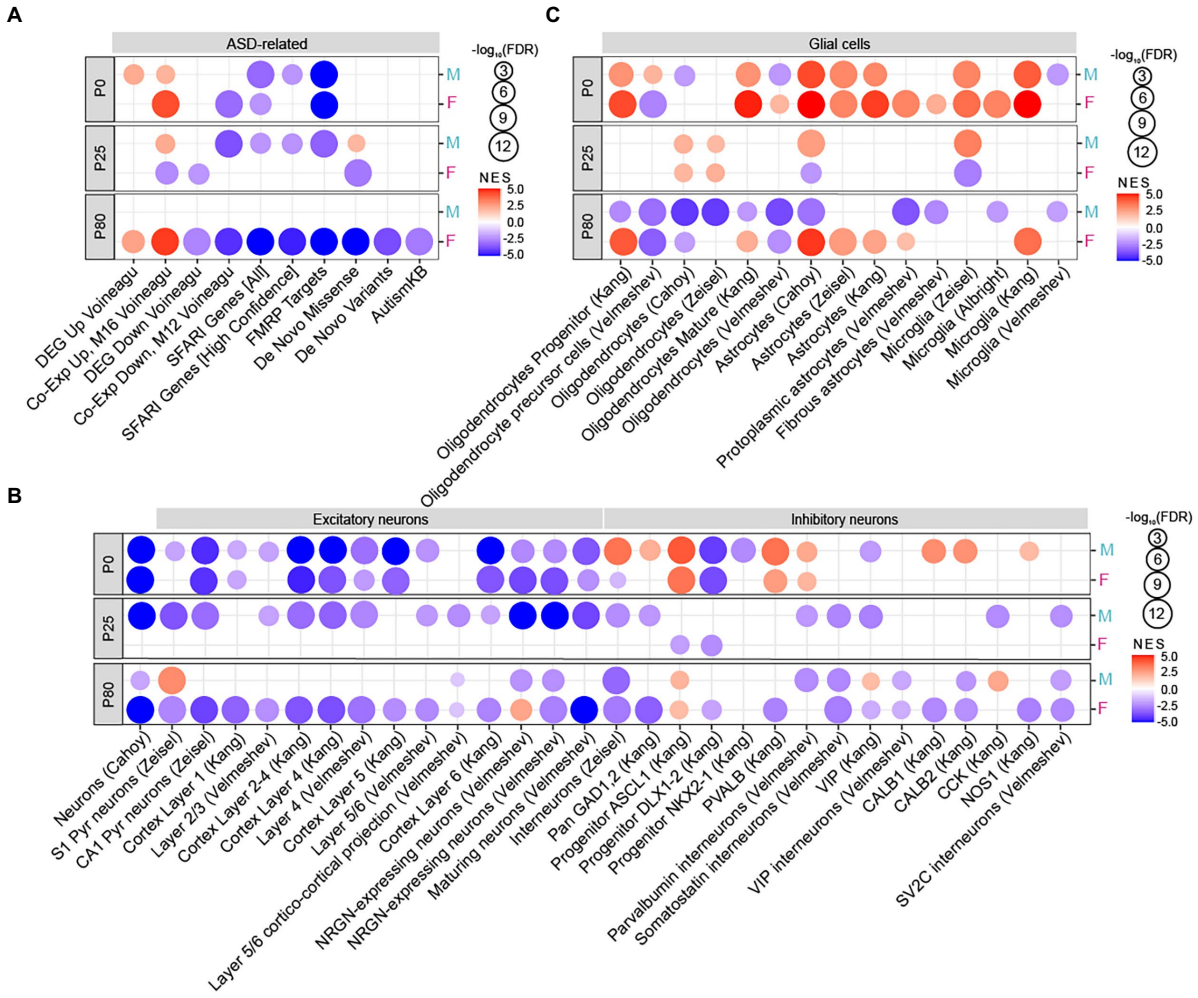
Age-differential ASD-like transcriptomic changes in adult Chd8+/S62X males and females. **(A)** GSEA of P0, P25, and P80 male and female HT/WT transcripts for ASD-related/risk gene sets, including those upregulated in ASD (DEG_Up_Voineagu and Co-Exp_Up_M16_Voineagu) and downregulated in ASD (DEG_Down_Voineagu and Co-Exp_Down_M12_Voineagu) as well as ASD-risk gene sets that are likely downregulated in ASD (SFARI [all], SFARI [high confidence], FMRP targets, DeNovoMis, DeNovoVariants, and AutismKB). (see Supplementary Tables S5–S7 for full results). (*n* = 3 mice for P0/25/80, male/female, and WT/HT mice, FDR < 0.05). **(B)** GSEA of P0, P25, and P80 male and female HT/WT transcripts for cell-type–specific gene sets related to neurons (cortical layers and GABA sub-types). (*n* = 3 mice for P0/25/80, male/female, and WT/HT mice, FDR < 0.05). **(C)** GSEA of P0, P25, and P80 male and female HT/WT transcripts for cell-type–specific gene sets related to glial cells (oligodendrocytes, astrocytes, and microglia; *n* = 3 mice for P0/25/80, male/female, and WT/HT mice, FDR < 0.05).

GSEA results revealed that, at P0, both male and female *Chd8^+/S62X^* mice showed transcriptomic changes similar to those that occur in ASD (hereafter termed ‘ASD-like pattern’). Specifically, P0 male HT/WT transcripts—the complete set of transcripts differentially expressed between heterozygous *Chd8^+/S62X^* mice and WT mice (ranked by fold-change signs x –log10 [*p*-value])—were positively enriched for ASD-related gene sets (summarized in Supplementary Tables S4, S5) that are upregulated in ASD (DEG_Up_Voineagu and Co_Exp_Up_Voineagu; Voineagu et al., 2011; Werling et al., 2016), although not negatively enriched for ASD-downregulated gene sets (DEG_Down_Voineagu and Co_Exp_Down_Voineagu; Figure 4A).

In addition, P0 male HT/WT transcripts showed negative enrichments for ASD-risk gene sets such as SFARI genes^2^ and FMRP targets (Werling et al., 2016) but not for DeNovoMis (protein-disrupting or missense rare *de novo* variants; Iossifov et al., 2014; Werling et al., 2016), DeNovoVariants (protein-disrupting rare *de novo* variants; Iossifov et al., 2014; Werling et al., 2016), or AutismKB (Autism KnowledgeBase; Yang et al., 2018; Figure 4A); these gene sets are thought to be downregulated by ASD-related deletion, non-sense, missense, frame-shift, and/or splice-site mutations. P0 female HT/WT transcripts showed positive and negative enrichments in ASD-related and ASD-risk gene sets. This is similar to the changes observed in P0-male HT/WT transcripts, which indicates comparable transcriptomic changes in P0 mutant males and females.

In contrast, P25 male and female HT/WT transcripts showed distinct enrichment patterns. P25 males showed an “ASD-like” pattern similar to that observed in ASD as well as P0 males and females, whereas P25 females showed a pattern that is largely unrelated to ASD, with minimal enrichments for ASD-related/risk gene sets (Figure 4A; Supplementary Table S6).

Intriguingly, at P80, male HT/WT transcripts showed minimal enrichment for ASD-related/risk gene sets, whereas female HT/WT transcripts displayed a strong ASD-like pattern (Figure 4A; Supplementary Table S7). These results indicate that mutant male transcriptomes show strong ASD-like, monophasic patterns at ~P0 and P25 but not at P80, whereas mutant female transcriptomes show strong ASD-like patterns at ~P0 and P80 (a biphasic pattern), with a weak or no ASD-like pattern at ~P25.

Previous studies have also reported that ASD is associated with cell-type–specific transcriptomic changes, including decreased gene expression in neurons and oligodendrocytes and increased gene expression in astrocytes and microglia (Voineagu et al., 2011; Werling et al., 2016), although alternative splicing of these genes controlled by proteins, including RBFOX1, can provide a distinct layer of regulation (Irimia et al., 2014; Parikshak et al., 2016; Quesnel-Vallieres et al., 2019). Tests of *Chd8^+/S62X^* transcriptomes for the enrichment of cell-type-specific gene sets (Albright and Gonzalez-Scarano, 2004; Cahoy et al., 2008; Kang et al., 2011; Zeisel et al., 2015; Werling et al., 2016; Velmeshev et al., 2019, 2020) showed that both male and female P0 HT/WT transcriptomes were negatively enriched for excitatory neuron-related gene sets but positively enriched for astrocyte-and microglia-related gene sets (Figures 4B,C), consistent with “ASD-like” patterns. However, mixed positive and negative enrichments could also be observed for some gene sets associated with inhibitory neurons and oligodendrocytes, standing in between the ASD-like pattern and an opposite change (hereafter termed “reverse-ASD-like” pattern).

P25 male HT/WT transcripts maintained this tendency toward an ASD-like pattern, with the negative enrichment for inhibitory neurons getting stronger, but P25 female HT/WT transcripts showed a pattern opposite that of the ASD-like pattern (i.e., nearly undetectable neuronal enrichments, upregulated oligodendrocyte genes, and downregulated astrocyte and microglial genes; Figures 4B,C). At P80, male HT/WT transcripts showed enrichment patterns that were partly ASD-like (moderately downregulated neuronal genes and downregulated oligodendrocyte genes) and reverse-ASD (downregulated astrocyte and microglia genes), whereas female HT/WT transcripts showed a strong ASD-like pattern (downregulated neuronal and oligodendrocyte genes and upregulated astrocyte and microglia genes).

These results collectively suggest that male HT/WT transcripts show monophasic ASD-like transcriptomic changes that peak at ~P0 and P25, whereas female HT/WT transcripts show biphasic transcriptomic changes peaking at ~P0 and P80.

### Age-differential functional changes in male and female *Chd8^+/S62X^* transcriptomes

We next tested whether *Chd8^+/S62X^* transcriptomes are enriched for specific biological functions by applying GSEA to gene sets in the Gene Ontology (C5) gene-set database (currently containing ~15,700 gene sets; http://www.gsea-msigdb.org/gsea/msigdb).

**Figure 5:**
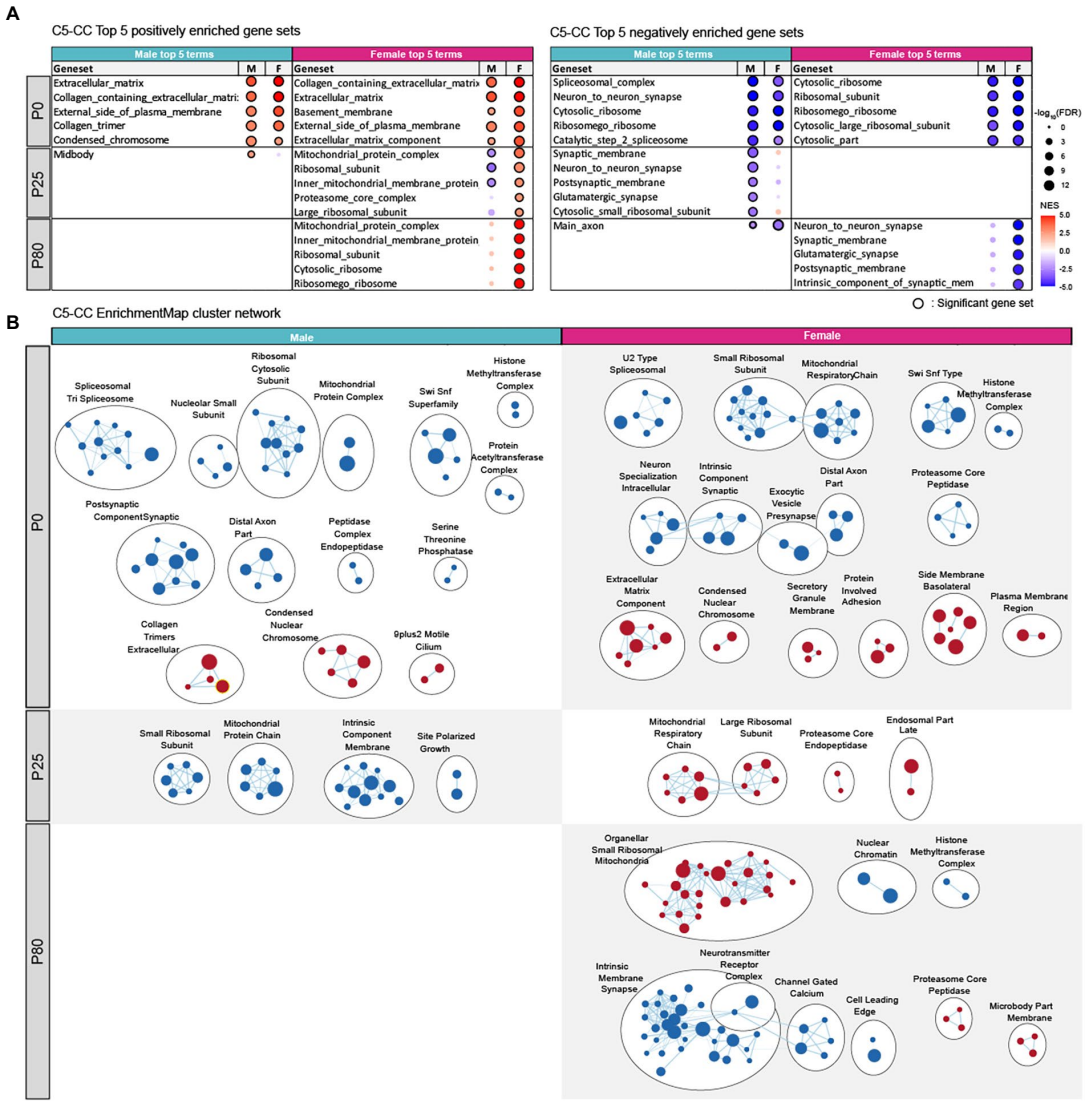
Biological functions differentially altered in male and female *Chd8^+/S62X^* transcriptomes. **(A)** GSEA of P0, P25, and P80 male and female HT/WT transcripts for gene sets in the cellular components (CC) domain of the C5 database (http://software.broadinstitute.org/gsea). Top-five gene sets positively (red circles) or negatively (blue circles) enriched in male or female transcripts are listed, together with the same gene sets in the other sex and their enrichment levels (see Supplementary Tables S5–S7 for full results). (*n* = 3 mice for P0/P25/P80 male/female mice, FDR (false discovery rate) < 0.05; NES, normalized enrichment score). **(B)** Integration and visualization of enriched genes in larger clusters using Cytoscape EnrichmentMap. (*n* = 3 mice for P0/25/80 male/female mice; node cutoff, FDR < 0.05 and *p* < 0.001; edge cutoff, overlap coefficient > 0.5).

In the cellular component (CC) domain, P0 male HT/WT transcripts were positively enriched for extracellular matrix-and chromatin-related gene sets, as shown by the top-five gene-set list (Figure 5A; see Supplementary Tables S5–S7 for full GSEA results [GSEA-CC]) and the clusters of enriched gene sets revealed by the EnrichmentMap Cytoscape App (Isserlin et al., 2014; Figure 5B). P0 male HT/WT transcripts were also negatively enriched for synapse-, spliceosome-, ribosome-, mitochondria-, and chromatin remodeling-related gene sets, as shown by top-five and clustered gene sets (Figures 5A,B). P0-female-HT/WT transcripts showed similar positive and negative enrichment patterns.

At P25, male HT/WT transcripts were negatively enriched for synapse-, ribosome-, and mitochondria-related gene sets (Figures 5A,B), a pattern with some similarity to that observed in P0 males. In P25 females, however, HT/WT transcripts were positively enriched for ribosome-and mitochondria-related gene sets (Figures 5A,B), a pattern largely opposite that observed in P25 males.

At P80, male HT/WT transcripts showed minimal enrichment patterns (positive or negative), whereas female HT/WT transcripts showed strong negative enrichments for synapse-related gene sets and positive enrichments for ribosome/mitochondria-related gene sets (Figures 5A,B).

**Figure 6:**
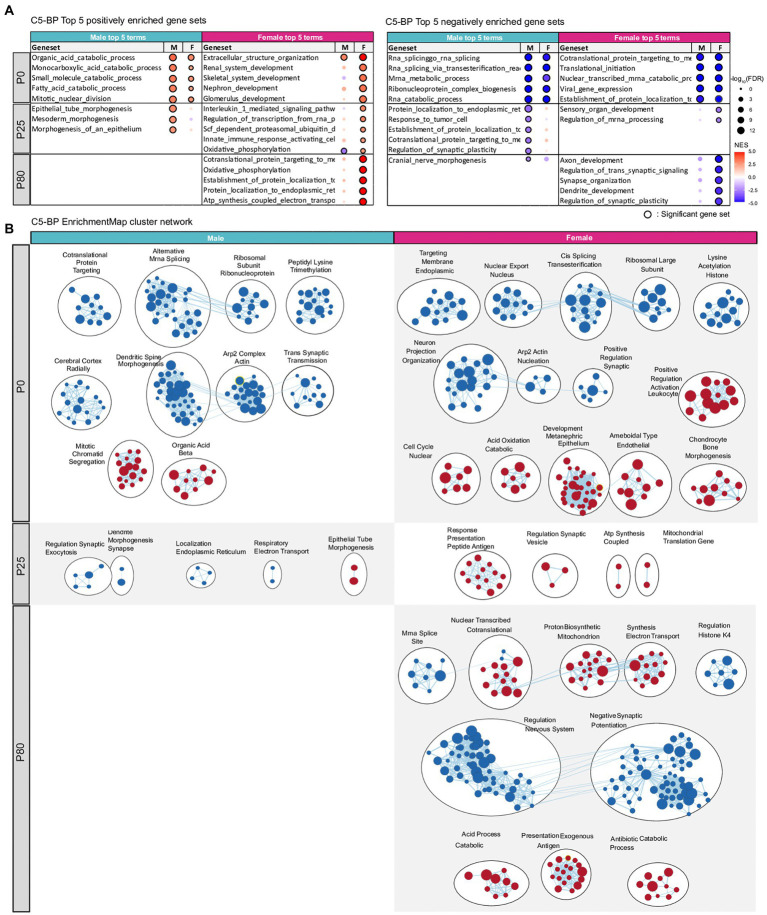
Transcriptomic changes in *Chd8^+/S62X^* males and females for gene sets in the biological process domain. **(A)** GSEA of P0, P25, and P80 male and female HT/WT transcripts for gene sets in the biological process (BP) domain of the C5 database (http://software.broadinstitute.org/gsea). Top-five gene sets positively or negatively enriched in male or female transcripts are listed, together with the same gene sets in the other sex and their enrichment levels (see Supplementary Tables S5–S7 for complete results). (*n* = 3 mice for P0/P25/P80 male/female mice, FDR (false discovery rate) < 0.05; NES, normalized enrichment score). **(B)** Integration and visualization of enriched genes in larger clusters using Cytoscape EnrichmentMap. (*n* = 3 mice for P0/25/80 male/female mice; node cutoff, FDR < 0.05 and *p* < 0.001; edge cutoff, overlap coefficient > 0.5).

Tests of gene sets in biological process and molecular function domains (C5 database) revealed enrichment patterns that are largely similar to those observed for CC-domain gene sets, although MF-domain gene sets tended to show weaker enrichments (Figure 6A,B; Supplementary Figure S1; Supplementary Tables S5–S7).

These results collectively suggest that synapse genes are downregulated at P0 and P25 in mutant males and at P0 and P80 in mutant females. Notably, downregulations of synapse genes in P25 males and P80 females were strongly correlated with the ASD-like transcriptomic patterns (Voineagu et al., 2011; Werling et al., 2016) observed in these animals.

## Discussion

We characterized synaptic and transcriptomic phenotypes of *Chd8^+/S62X^* males and females at multiple postnatal stages and found age-differential sexual dimorphism at both synaptic and transcriptomic levels.

Our previous study *Chd8^+/S62X^* mice revealed that behavioral deficits are observed in male but not female juveniles, whereas adult mutant males and females display similar behavioral deficits (Lee et al., 2022). This age-differential sexual dimorphism in the behaviors of *Chd8^+/S62X^* mice differs from the persistent male-preponderant behavioral deficits in *Chd8^+/N2373X^* pups, juveniles, and adults (Jung et al., 2018). What might be the mechanisms underlying the age-differential behavioral deficits in *Chd8^+/S62X^* males and females?

Our results indicated decreased excitatory synaptic transmission in male *Chd8^+/S62X^* juveniles. This change was correlated with both ASD-like transcriptomic changes and autistic-like behaviors (enhanced mother seeking/attachment), suggesting that the decreased excitatory transmission contributes to the behavioral deficits. In contrast, female juvenile mutant mice showed increased excitatory synaptic transmission that correlated with considerably weakened ASD-like transcriptomic patterns (relative to newborn-stage patterns) and largely normal behaviors. Therefore, excitatory synaptic suppression likely underlies the male-preponderant behavioral deficits in juvenile mutant mice. This hypothesis is in line with the synaptic dysfunction frequently implicated in ASD (Bourgeron, 2015), active synaptic development at juvenile stages (i.e., 2–3 postnatal weeks; Sheng and Sala, 2001), and neuronal/synaptic excitation/inhibition imbalances implicated in ASD (Nelson and Valakh, 2015; Lee et al., 2017). Notably, overproduction of inhibitory neurons, which would also induce a decrease in the excitation/inhibition ratio, similar to our results (glutamate synaptic suppression), was observed in human ASD-related cortical organoids (Mariani et al., 2015), suggesting that distinct mechanisms in mice and humans may underlie a similar excitation/inhibition imbalance.

At adult stages, *Chd8^+/S62X^* males and females show strong and shared autistic-like behavioral deficits (self-grooming and anxiety-like behaviors; Lee et al., 2022). These mice also exhibit shared increases in excitatory synaptic transmission (mEPSC results). These strong correlations suggest that the increased excitatory synaptic transmission may underlie autistic-like behaviors in mutant males and females at adult stages. While displaying similar increases in excitatory synaptic transmission as adults, *Chd8^+/S62X^* males and females seem to undergo distinct synaptic and transcriptomic changes across the juvenile-to-adult temporal axis. Specifically, *Chd8^+/S62X^* males show decreased excitatory synaptic transmission as juveniles but increased excitatory transmission as adults. This age-dependent reversal could reflect the consequence of excessive compensatory changes to normalize the decreased juvenile excitatory synaptic transmission. Such compensatory changes and synaptic normalization may induce the substantial weakening of ASD-like transcriptomic patterns that occurs between juvenile and adult stages, although these compensations appear to be not sufficient to rescue the behavioral deficits.

Adult female *Chd8^+/S62X^* mice, unlike adult males, show excitatory synaptic transmission that continues to be increased across juvenile and adult stages. However, the weakened ASD-like transcriptomic patterns in juvenile female mutant mice, relative to newborn mice, become strong again in adult female mutant mice in a biphasic manner, similar to the ASD-like transcriptomic patterns in newborn mice. It is possible that the increased excitatory synaptic transmission, which likely rescues the behavioral deficits in juvenile mutant females, may no longer be able to protect adult females from developing behavioral deficits. Notably, in adult mutant females, the increased excitatory synaptic transmission in adult mutant females is sensitive to network compensation, as supported by the adult sEPSC results, unlike the increased excitatory synaptic transmission in juvenile mutant females that is resistant to network compensation (juvenile sEPSC results). This may represent acute adult-onset compensatory changes aiming to normalize excitatory synaptic transmission, which may be mediated by the strongly suppressed synaptic gene expressions, although this might induce secondary synapse-related changes that aggravate brain functions and behaviors.

The data collected from *Chd8^+/S62X^* mice in the present and previous studies are limited by the lack of causal relationships between the observed molecular, synaptic, and behavioral changes. Although additional details remain to be determined, our data may provide some baseline information given that little is known about (1) whether the strong male preponderance in autistic individuals with *CHD8* mutations could be recapitulated in mice, (2) whether different *CHD8* mutations lead to heterogeneous male–female differences in mice in a spatiotemporally differential manner, and (3) what mechanisms underlie the *CHD8*-related sexual dimorphism and the general sexual dimorphism widespread in ASD and neurodevelopmental and psychiatric disorders.

In summary, our results provide evidence suggesting that the CHD8-S62X mutation derived from an autistic individual leads to age-differential and sexually dimorphic synaptic and transcriptomic changes in mice.

## Data availability statement

The RNA-Seq results are deposited in the NCBI GEO (National Center for Biotechnology Information, Gene Expression Omnibus) repository as GSE167053..

## Ethics statement

The animal study was reviewed and approved by Committee on Animal Research at KAIST.

## Author contributions

SL and HKw performed electrophysiological experiments. SL and HKa performed RNA-Seq analyses. HKa and EK wrote the manuscript. All authors contributed to the article and approved the submitted version.

## Funding

This work was supported by the IBS-R002-D1 to EK.

## Conflict of interest

The authors declare that the research was conducted in the absence of any commercial or financial relationships that could be construed as a potential conflict of interest.

## Publisher’s note

All claims expressed in this article are solely those of the authors and do not necessarily represent those of their affiliated organizations, or those of the publisher, the editors and the reviewers. Any product that may be evaluated in this article, or claim that may be made by its manufacturer, is not guaranteed or endorsed by the publisher.

## Footnotes

^1^ https://www.gsea-msigdb.org/gsea/index.jsp

^2^ https://gene.sfari.org/

## References

[ref1] Albright A. V. Gonzalez-Scarano F. (2004). Microarray analysis of activated mixed glial (microglia) and monocyte-derived macrophage gene expression. J. Neuroimmunol. 157, 27–38. doi: 10.1016/j.jneuroim.2004.09.007, PMID: 15579277

[ref2] Andreae L. C. Basson M. A. (2018). Sex bias in autism: new insights from Chd8 mutant mice? Nat. Neurosci. 21, 1144–1146. doi: 10.1038/s41593-018-0217-y, PMID: 30127425

[ref3] Barnard R. A. Pomaville M. B. O'Roak B. J. (2015). Mutations and modeling of the chromatin remodeler CHD8 define an emerging autism etiology. Front. Neurosci. 9:477. doi: 10.3389/fnins.2015.00477, PMID: 26733790 PMC4681771

[ref4] Bernier R. Golzio C. Xiong B. Stessman H. A. Coe B. P. Penn O.. (2014). Disruptive CHD8 mutations define a subtype of autism early in development. Cells 158, 263–276. doi: 10.1016/j.cell.2014.06.017, PMID: 24998929 PMC4136921

[ref5] Bourgeron T. (2015). From the genetic architecture to synaptic plasticity in autism spectrum disorder. Nat. Rev. Neurosci. 16, 551–563. doi: 10.1038/nrn3992, PMID: 26289574

[ref6] Breuss M. W. Gleeson J. G. (2016). When size matters: CHD8 in autism. Nat. Neurosci. 19, 1430–1432. doi: 10.1038/nn.4431, PMID: 27786184

[ref7] Cahoy J. D. Emery B. Kaushal A. Foo L. C. Zamanian J. L. Christopherson K. S.. (2008). A transcriptome database for astrocytes, neurons, and oligodendrocytes: a new resource for understanding brain development and function. J. Neurosci. 28, 264–278. doi: 10.1523/JNEUROSCI.4178-07.2008, PMID: 18171944 PMC6671143

[ref8] Chen X. Chen T. Dong C. Chen H. Dong X. Yang L.. (2022). Deletion of CHD8 in cerebellar granule neuron progenitors leads to severe cerebellar hypoplasia, ataxia, and psychiatric behavior in mice. J. Genet. Genomics 49, 859–869. doi: 10.1016/j.jgg.2022.02.011, PMID: 35231638

[ref9] Cherepanov S. M. Gerasimenko M. Yuhi T. Furuhara K. Tsuji C. Yokoyama S.. (2021). Oxytocin ameliorates impaired social behavior in a Chd8 haploinsufficiency mouse model of autism. BMC Neurosci. 22:32. doi: 10.1186/s12868-021-00631-6, PMID: 33933000 PMC8088024

[ref10] Coakley-Youngs E. Ranatunga M. Richardson S. Getti G. Shorter S. Fivaz M. (2022). Autism-associated CHD8 keeps proliferation of human neural progenitors in check by lengthening the G1 phase of the cell cycle. Biol. Open 11:bio058941. doi: 10.1242/bio.058941, PMID: 36222238 PMC9548376

[ref11] Cotney J. Muhle R. A. Sanders S. J. Liu L. Willsey A. J. Niu W.. (2015). The autism-associated chromatin modifier CHD8 regulates other autism risk genes during human neurodevelopment. Nat. Commun. 6:6404. doi: 10.1038/ncomms7404, PMID: 25752243 PMC4355952

[ref12] Ding S. Lan X. Meng Y. Yan C. Li M. Li X.. (2021). CHD8 safeguards early neuroectoderm differentiation in human ESCs and protects from apoptosis during neurogenesis. Cell Death Dis. 12:981. doi: 10.1038/s41419-021-04292-5, PMID: 34686651 PMC8536677

[ref13] Dingemans A. J. M. Truijen K. M. G. van de Ven S. Bernier R. Bongers E. Bouman A.. (2022). The phenotypic spectrum and genotype-phenotype correlations in 106 patients with variants in major autism gene CHD8. Transl. Psychiatry 12:421. doi: 10.1038/s41398-022-02189-1, PMID: 36182950 PMC9526704

[ref14] Dong C. Zhao C. Chen X. Berry K. Wang J. Zhang F.. (2022). Conserved and distinct functions of the autism-related chromatin remodeler CHD8 in embryonic and adult forebrain neurogenesis. J. Neurosci. 42, 8373–8392. doi: 10.1523/JNEUROSCI.2400-21.2022, PMID: 36127134 PMC9653284

[ref15] Durak O. Gao F. Kaeser-Woo Y. J. Rueda R. Martorell A. J. Nott A.. (2016). Chd8 mediates cortical neurogenesis via transcriptional regulation of cell cycle and Wnt signaling. Nat. Neurosci. 19, 1477–1488. doi: 10.1038/nn.4400, PMID: 27694995 PMC5386887

[ref16] Ellingford R. A. Panasiuk M. J. de Meritens E. R. Shaunak R. Naybour L. Browne L.. (2021). Cell-type-specific synaptic imbalance and disrupted homeostatic plasticity in cortical circuits of ASD-associated Chd8 haploinsufficient mice. Mol. Psychiatry 26, 3614–3624. doi: 10.1038/s41380-021-01070-9, PMID: 33837267 PMC8505247

[ref17] Gompers A. L. Su-Feher L. Ellegood J. Copping N. A. Riyadh M. A. Stradleigh T. W.. (2017). Germline Chd8 haploinsufficiency alters brain development in mouse. Nat. Neurosci. 20, 1062–1073. doi: 10.1038/nn.4592, PMID: 28671691 PMC6008102

[ref18] Haddad Derafshi B. Danko T. Chanda S. Batista P. J. Litzenburger U. Lee Q. Y.. (2022). The autism risk factor CHD8 is a chromatin activator in human neurons and functionally dependent on the ERK-MAPK pathway effector ELK1. Sci. Rep. 12:22425. doi: 10.1038/s41598-022-23614-x, PMID: 36575212 PMC9794786

[ref19] Hayot G. Massonot M. Keime C. Faure E. Golzio C. (2023). Loss of autism-candidate CHD8 perturbs neural crest development and intestinal homeostatic balance. Life Sci. Alliance 6:e202201456. doi: 10.26508/lsa.202201456 36375841 PMC9664244

[ref20] Hulbert S. W. Wang X. Gbadegesin S. O. Xu Q. Xu X. Jiang Y. H. (2020). A novel Chd8 mutant mouse displays altered ultrasonic vocalizations and enhanced motor coordination. Autism Res. 13, 1685–1697. doi: 10.1002/aur.2353, PMID: 32815320 PMC7780289

[ref21] Hurley S. Mohan C. Suetterlin P. Ellingford R. Riegman K. L. H. Ellegood J.. (2021). Distinct, dosage-sensitive requirements for the autism-associated factor CHD8 during cortical development. Mol. Autism. 12:16. doi: 10.1186/s13229-020-00409-3, PMID: 33627187 PMC7905672

[ref22] Iossifov I. O'Roak B. J. Sanders S. J. Ronemus M. Krumm N. Levy D.. (2014). The contribution of de novo coding mutations to autism spectrum disorder. Nature 515, 216–221. doi: 10.1038/nature13908, PMID: 25363768 PMC4313871

[ref23] Irimia M. Weatheritt R. J. Ellis J. D. Parikshak N. N. Gonatopoulos-Pournatzis T. Babor M.. (2014). A highly conserved program of neuronal microexons is misregulated in autistic brains. Cells 159, 1511–1523. doi: 10.1016/j.cell.2014.11.035, PMID: 25525873 PMC4390143

[ref24] Isserlin R. Merico D. Voisin V. Bader G. D. (2014). Enrichment map—a Cytoscape app to visualize and explore OMICs pathway enrichment results. F1000Res 3:141. doi: 10.12688/f1000research.4536.1 25075306 PMC4103489

[ref25] Jimenez J. A. Ptacek T. S. Tuttle A. H. Schmid R. S. Moy S. S. Simon J. M.. (2020). Chd8 haploinsufficiency impairs early brain development and protein homeostasis later in life. Mol. Autism. 11:74. doi: 10.1186/s13229-020-00369-8, PMID: 33023670 PMC7537101

[ref26] Jimenez J. A. Simon J. M. Hu W. Moy S. S. Harper K. M. Liu C. W.. (2022). Developmental pyrethroid exposure and age influence phenotypes in a Chd8 haploinsufficient autism mouse model. Sci. Rep. 12:5555. doi: 10.1038/s41598-022-09533-x, PMID: 35365720 PMC8975859

[ref27] Jung H. Park H. Choi Y. Kang H. Lee E. Kweon H.. (2018). Sexually dimorphic behavior, neuronal activity, and gene expression in Chd8-mutant mice. Nat. Neurosci. 21, 1218–1228. doi: 10.1038/s41593-018-0208-z, PMID: 30104731

[ref28] Kang H. J. Kawasawa Y. I. Cheng F. Zhu Y. Xu X. Li M.. (2011). Spatio-temporal transcriptome of the human brain. Nature 478, 483–489. nature10523 [pii]. doi: 10.1038/nature10523, PMID: 22031440 PMC3566780

[ref29] Katayama Y. Nishiyama M. Shoji H. Ohkawa Y. Kawamura A. Sato T.. (2016). CHD8 haploinsufficiency results in autistic-like phenotypes in mice. Nature 537, 675–679. doi: 10.1038/nature19357, PMID: 27602517

[ref30] Kawamura A. Katayama Y. Kakegawa W. Ino D. Nishiyama M. Yuzaki M.. (2021). The autism-associated protein CHD8 is required for cerebellar development and motor function. Cell Rep. 35:108932. doi: 10.1016/j.celrep.2021.108932, PMID: 33826902

[ref31] Kerschbamer E. Arnoldi M. Tripathi T. Pellegrini M. Maturi S. Erdin S.. (2022). CHD8 suppression impacts on histone H3 lysine 36 trimethylation and alters RNA alternative splicing. Nucleic Acids Res. 50, 12809–12828. doi: 10.1093/nar/gkac1134, PMID: 36537238 PMC9825192

[ref32] Kweon H. Jung W. B. Im G. H. Ryoo J. Lee J. H. Do H.. (2021). Excitatory neuronal CHD8 in the regulation of neocortical development and sensory-motor behaviors. Cell Rep. 34:108780. doi: 10.1016/j.celrep.2021.108780, PMID: 33626347

[ref33] Lee S. Y. Kweon H. Kang H. Kim E. (2022). Age-differential sexual dimorphism in CHD8-S62X-mutant mouse behaviors. Front. Mol. Neurosci. 15:1022306. doi: 10.3389/fnmol.2022.1022306, PMID: 36385756 PMC9641250

[ref34] Lee E. Lee J. Kim E. (2017). Excitation/inhibition imbalance in animal models of autism Spectrum disorders. Biol. Psychiatry 81, 838–847. doi: 10.1016/j.biopsych.2016.05.011, PMID: 27450033

[ref35] Love M. I. Huber W. Anders S. (2014). Moderated estimation of fold change and dispersion for RNA-seq data with DESeq2. Genome Biol. 15:550. doi: 10.1186/s13059-014-0550-8, PMID: 25516281 PMC4302049

[ref36] Mariani J. Coppola G. Zhang P. Abyzov A. Provini L. Tomasini L.. (2015). FOXG1-dependent dysregulation of GABA/glutamate neuron differentiation in autism Spectrum disorders. Cells 162, 375–390. doi: 10.1016/j.cell.2015.06.034, PMID: 26186191 PMC4519016

[ref37] Merner N. Forgeot d'Arc B. Bell S. C. Maussion G. Peng H. Gauthier J.. (2016). A de novo frameshift mutation in chromodomain helicase DNA-binding domain 8 (CHD8): a case report and literature review. Am. J. Med. Genet. A 170, 1225–1235. doi: 10.1002/ajmg.a.37566 26789910

[ref38] Nelson S. B. Valakh V. (2015). Excitatory/inhibitory balance and circuit homeostasis in autism Spectrum disorders. Neuron 87, 684–698. doi: 10.1016/j.neuron.2015.07.033, PMID: 26291155 PMC4567857

[ref39] O'Roak B. J. Vives L. Fu W. Egertson J. D. Stanaway I. B. Phelps I. G.. (2012). Multiplex targeted sequencing identifies recurrently mutated genes in autism spectrum disorders. Science 338, 1619–1622. doi: 10.1126/science.1227764, PMID: 23160955 PMC3528801

[ref40] Parikshak N. N. Swarup V. Belgard T. G. Irimia M. Ramaswami G. Gandal M. J.. (2016). Genome-wide changes in lncRNA, splicing, and regional gene expression patterns in autism. Nature 540, 423–427. doi: 10.1038/nature20612, PMID: 27919067 PMC7102905

[ref41] Patro R. Duggal G. Love M. I. Irizarry R. A. Kingsford C. (2017). Salmon provides fast and bias-aware quantification of transcript expression. Nat. Methods 14, 417–419. doi: 10.1038/nmeth.4197, PMID: 28263959 PMC5600148

[ref42] Paulsen B. Velasco S. Kedaigle A. J. Pigoni M. Quadrato G. Deo A. J.. (2022). Autism genes converge on asynchronous development of shared neuron classes. Nature 602, 268–273. doi: 10.1038/s41586-021-04358-6, PMID: 35110736 PMC8852827

[ref43] Platt R. J. Zhou Y. Slaymaker I. M. Shetty A. S. Weisbach N. R. Kim J. A.. (2017). Chd8 mutation leads to autistic-like behaviors and impaired striatal circuits. Cell Rep. 19, 335–350. doi: 10.1016/j.celrep.2017.03.052, PMID: 28402856 PMC5455342

[ref44] Quesnel-Vallieres M. Weatheritt R. J. Cordes S. P. Blencowe B. J. (2019). Autism spectrum disorder: insights into convergent mechanisms from transcriptomics. Nat. Rev. Genet. 20, 51–63. doi: 10.1038/s41576-018-0066-2, PMID: 30390048

[ref45] Schumann C. M. Hamstra J. Goodlin-Jones B. L. Lotspeich L. J. Kwon H. Buonocore M. H.. (2004). The amygdala is enlarged in children but not adolescents with autism; the hippocampus is enlarged at all ages. J. Neurosci. 24, 6392–6401. doi: 10.1523/JNEUROSCI.1297-04.2004, PMID: 15254095 PMC6729537

[ref46] Sheng M. Sala C. (2001). PDZ domains and the organization of supramolecular complexes. Annu. Rev. Neurosci. 24, 1–29. doi: 10.1146/annurev.neuro.24.1.1, PMID: 11283303

[ref47] Soneson C. Love M. I. Robinson M. D. (2015). Differential analyses for RNA-seq: transcript-level estimates improve gene-level inferences. F1000Res 4:1521. doi: 10.12688/f1000research.7563.2 26925227 PMC4712774

[ref48] Sood S. Weber C. M. Hodges H. C. Krokhotin A. Shalizi A. Crabtree G. R. (2020). CHD8 dosage regulates transcription in pluripotency and early murine neural differentiation. Proc. Natl. Acad. Sci. U. S. A. 117, 22331–22340. doi: 10.1073/pnas.1921963117, PMID: 32839322 PMC7486765

[ref49] Stessman H. A. Xiong B. Coe B. P. Wang T. Hoekzema K. Fenckova M.. (2017). Targeted sequencing identifies 91 neurodevelopmental-disorder risk genes with autism and developmental-disability biases. Nat. Genet. 49, 515–526. doi: 10.1038/ng.3792, PMID: 28191889 PMC5374041

[ref50] Subramanian A. Tamayo P. Mootha V. K. Mukherjee S. Ebert B. L. Gillette M. A.. (2005). Gene set enrichment analysis: a knowledge-based approach for interpreting genome-wide expression profiles. Proc. Natl. Acad. Sci. U. S. A. 102, 15545–15550. doi: 10.1073/pnas.0506580102, PMID: 16199517 PMC1239896

[ref51] Suetterlin P. Hurley S. Mohan C. Riegman K. L. H. Pagani M. Caruso A.. (2018). Altered neocortical gene expression, brain overgrowth and functional over-connectivity in Chd8 Haploinsufficient mice. Cereb. Cortex 28, 2192–2206. doi: 10.1093/cercor/bhy058, PMID: 29668850 PMC6018918

[ref52] Sugathan A. Biagioli M. Golzio C. Erdin S. Blumenthal I. Manavalan P.. (2014). CHD8 regulates neurodevelopmental pathways associated with autism spectrum disorder in neural progenitors. Proc. Natl. Acad. Sci. U. S. A. 111, E4468–E4477. doi: 10.1073/pnas.1405266111, PMID: 25294932 PMC4210312

[ref53] Thudium S. Palozola K. L'Her E. Korb E. (2022). Identification of a transcriptional signature found in multiple models of ASD and related disorders. Genome Res. 32, 1642–1654. doi: 10.1101/gr.276591.122, PMID: 36104286 PMC9528985

[ref54] Tu Z. Fan C. Davis A. K. Hu M. Wang C. Dandamudi A.. (2022). Autism-associated chromatin remodeler CHD8 regulates erythroblast cytokinesis and fine-tunes the balance of rho GTPase signaling. Cell Rep. 40:111072. doi: 10.1016/j.celrep.2022.111072, PMID: 35830790 PMC9302451

[ref55] Velmeshev D. Magistri M. Mazza E. M. C. Lally P. Khoury N. D'Elia E. R.. (2020). Cell-type-specific analysis of molecular pathology in autism identifies common genes and pathways affected across neocortical regions. Mol. Neurobiol. 57, 2279–2289. doi: 10.1007/s12035-020-01879-5, PMID: 32008165

[ref56] Velmeshev D. Schirmer L. Jung D. Haeussler M. Perez Y. Mayer S.. (2019). Single-cell genomics identifies cell type-specific molecular changes in autism. Science 364, 685–689. doi: 10.1126/science.aav8130, PMID: 31097668 PMC7678724

[ref57] Villa C. E. Cheroni C. Dotter C. P. Lopez-Tobon A. Oliveira B. Sacco R.. (2022). CHD8 haploinsufficiency links autism to transient alterations in excitatory and inhibitory trajectories. Cell Rep. 39:110615. doi: 10.1016/j.celrep.2022.110615, PMID: 35385734

[ref58] Voineagu I. Wang X. Johnston P. Lowe J. K. Tian Y. Horvath S.. (2011). Transcriptomic analysis of autistic brain reveals convergent molecular pathology. Nature 474, 380–384. doi: 10.1038/nature10110, PMID: 21614001 PMC3607626

[ref59] Wade A. A. Lim K. Catta-Preta R. Nord A. S. (2018). Common CHD8 genomic targets contrast with model-specific transcriptional impacts of CHD8 Haploinsufficiency. Front. Mol. Neurosci. 11:481. doi: 10.3389/fnmol.2018.00481, PMID: 30692911 PMC6339895

[ref60] Wang P. Lin M. Pedrosa E. Hrabovsky A. Zhang Z. Guo W.. (2015). CRISPR/Cas9-mediated heterozygous knockout of the autism gene CHD8 and characterization of its transcriptional networks in neurodevelopment. Mol. Autism. 6:55. doi: 10.1186/s13229-015-0048-6, PMID: 26491539 PMC4612430

[ref61] Wang P. Mokhtari R. Pedrosa E. Kirschenbaum M. Bayrak C. Zheng D.. (2017). CRISPR/Cas9-mediated heterozygous knockout of the autism gene CHD8 and characterization of its transcriptional networks in cerebral organoids derived from iPS cells. Mol. Autism. 8:11. doi: 10.1186/s13229-017-0124-1, PMID: 28321286 PMC5357816

[ref62] Weissberg O. Elliott E. (2021). The mechanisms of CHD8 in neurodevelopment and autism Spectrum disorders. Genes 12:1133. doi: 10.3390/genes12081133, PMID: 34440307 PMC8393912

[ref63] Werling D. M. Parikshak N. N. Geschwind D. H. (2016). Gene expression in human brain implicates sexually dimorphic pathways in autism spectrum disorders. Nat. Commun. 7:10717. doi: 10.1038/ncomms10717, PMID: 26892004 PMC4762891

[ref64] Xu Q. Liu Y. Y. Wang X. Tan G. H. Li H. P. Hulbert S. W.. (2018). Autism-associated CHD8 deficiency impairs axon development and migration of cortical neurons. Mol. Autism. 9:65. doi: 10.1186/s13229-018-0244-2, PMID: 30574290 PMC6299922

[ref65] Yang C. Li J. Wu Q. Yang X. Huang A. Y. Zhang J.. (2018). AutismKB 2.0: A knowledgebase for the genetic evidence of autism spectrum disorder. Database 2018:bay106. doi: 10.1093/database/bay106, PMID: 30339214 PMC6193446

[ref66] Yu Y. Zhang B. Ji P. Zuo Z. Huang Y. Wang N.. (2022). Changes to gut amino acid transporters and microbiome associated with increased E/I ratio in Chd8(+/−) mouse model of ASD-like behavior. Nat. Commun. 13:1151. doi: 10.1038/s41467-022-28746-2, PMID: 35241668 PMC8894489

[ref67] Zeisel A. Munoz-Manchado A. B. Codeluppi S. Lonnerberg P. La Manno G. Jureus A.. (2015). Brain structure. Cell types in the mouse cortex and hippocampus revealed by single-cell RNA-seq. Science 347, 1138–1142. doi: 10.1126/science.aaa1934, PMID: 25700174

[ref68] Zhao C. Dong C. Frah M. Deng Y. Marie C. Zhang F.. (2018). Dual requirement of CHD8 for chromatin landscape establishment and histone methyltransferase recruitment to promote CNS myelination and repair. Dev. Cell 45:e758, 753–768.e8. doi: 10.1016/j.devcel.2018.05.022, PMID: 29920279 PMC6063525

[ref69] Zhou X. Feliciano P. Shu C. Wang T. Astrovskaya I. Hall J. B.. (2022). Integrating de novo and inherited variants in 42,607 autism cases identifies mutations in new moderate-risk genes. Nat. Genet. 54, 1305–1319. doi: 10.1038/s41588-022-01148-2, PMID: 35982159 PMC9470534

