# Supplementary material for: Age-differential sexual dimorphisms in CHD8-S62X-mutant mouse synapses and transcriptomes
Source: Front Mol Neurosci. 2023 Feb 16;16:1111388. doi: 10.3389/fnmol.2023.1111388 (PMC9978779; doi:10.3389/fnmol.2023.1111388)
Supplement: Supplementary file 8 [file Data_Sheet_1.PDF]

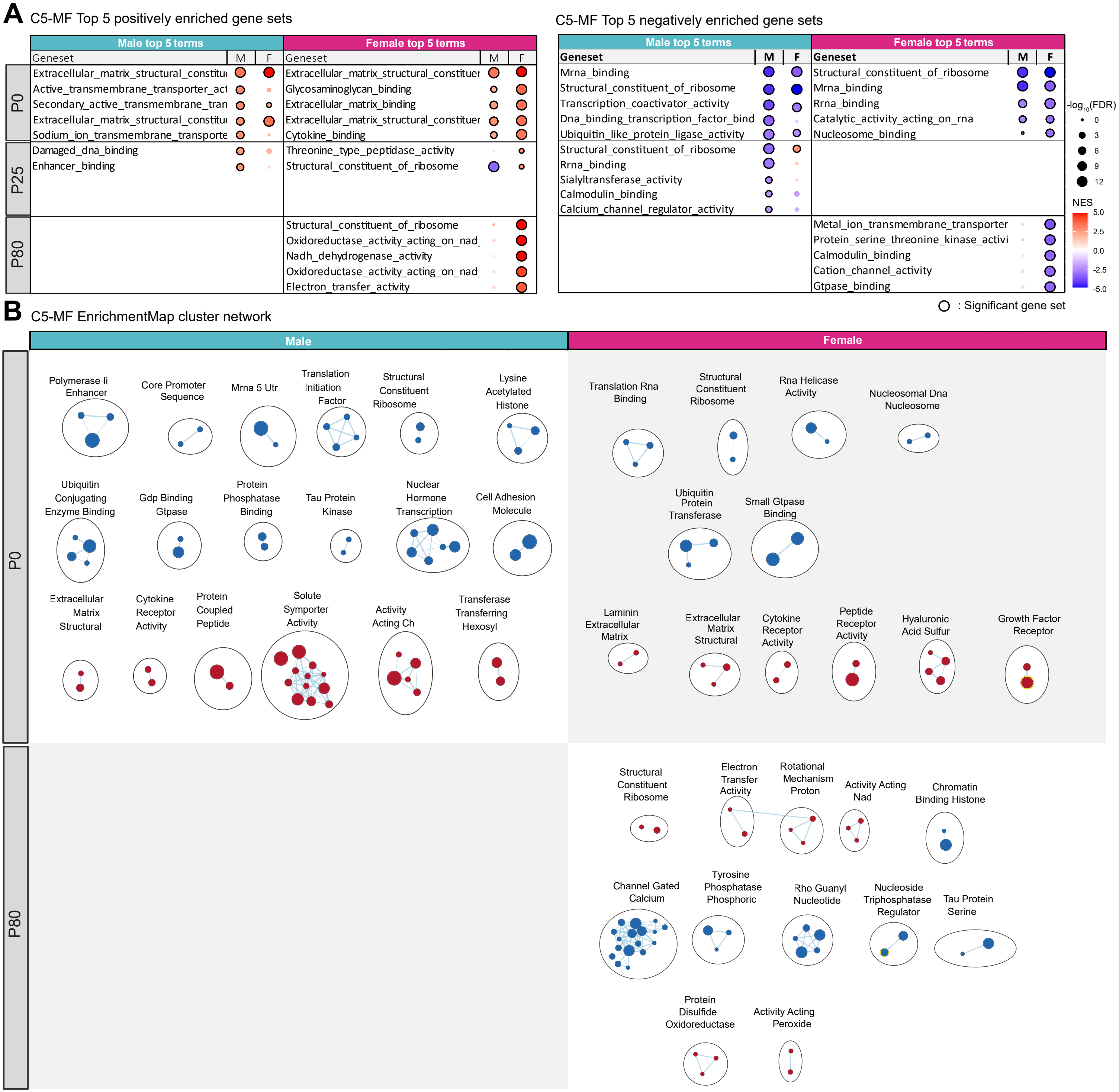

A
C5-MF Top 5 positively enriched gene sets
Male top 5 terms
Female top 5 terms
Geneset
M
F
P0
Extracellular_matrix_structural_constitu
Active_transmembrane_transporter_act
Secondary_active_transmembrane_tran
Extracellular_matrix_structural_constitu
Sodium_ion_transmembrane_transporte
Extracellular_matrix_structural_constituer
Glycosaminoglycan_binding
Extracellular_matrix_binding
Extracellular_matrix_structural_constituer
Cytokine_binding
P25
Damaged_dna_binding
Enhancer_binding
Threonine_type_peptidase_activity
Structural_constituent_of_ribosome
P80
Structural_constituent_of_ribosome
Oxidoreductase_activity_acting_on_nad_
Nadh_dehydrogenase_activity
Oxidoreductase_activity_acting_on_nad_
Electron_transfer_activity
C5-MF Top 5 negatively enriched gene sets
Male top 5 terms
Female top 5 terms
Geneset
M
F
Mrna_binding
Structural_constituent_of_ribosome
Transcription_coactivator_activity
Dna_binding_transcription_factor_bind
Ubiquitin_like_protein_ligase_activity
Structural_constituent_of_ribosome
Mrna_binding
Rrna_binding
Catalytic_activity_acting_on_rna
Nucleosome_binding
Structural_constituent_of_ribosome
Rrna_binding
Sialyltransferase_activity
Calmodulin_binding
Calcium_channel_regulator_activity
Metal_ion_transmembrane_transporter
Protein_serine_threonine_kinase_activi
Calmodulin_binding
Cation_channel_activity
Gtpase_binding
-log10(FDR)
0
3
6
9
12
NES
5.0
2.5
0.0
-2.5
-5.0
: Significant gene set
B
C5-MF EnrichmentMap cluster network
Male
Female
P0
Polymerase Ii Enhancer
Core Promoter Sequence
Mrna 5 Utr
Translation Initiation Factor
Structural Constituent Ribosome
Lysine Acetylated Histone
Ubiquitin Conjugating Enzyme Binding
Gdp Binding Gtpase
Protein Phosphatase Binding
Tau Protein Kinase
Nuclear Hormone Transcription
Cell Adhesion Molecule
Extracellular Matrix Structural
Cytokine Receptor Activity
Protein Coupled Peptide
Solute Symporter Activity
Activity Acting Ch
Transferase Transferring Hexosyl
Translation Rna Binding
Structural Constituent Ribosome
Rna Helicase Activity
Nucleosomal Dna Nucleosome
Ubiquitin Protein Transferase
Small Gtpase Binding
Laminin Extracellular Matrix
Extracellular Matrix Structural
Cytokine Receptor Activity
Peptide Receptor Activity
Hyaluronic Acid Sulfur
Growth Factor Receptor
P80
Structural Constituent Ribosome
Electron Transfer Activity
Rotational Mechanism Proton
Activity Acting Nad
Chromatin Binding Histone
Channel Gated Calcium
Tyrosine Phosphatase Phosphoric
Rho Guanyl Nucleotide
Nucleoside Triphosphatase Regulator
Tau Protein Serine
Protein Disulfide Oxidoreductase
Activity Acting Peroxide
